# Well-Ordered Bicontinuous Nanohybrids from a Bottom-Up Approach for Enhanced Strength and Toughness

**DOI:** 10.1021/acs.nanolett.4c03157

**Published:** 2024-08-28

**Authors:** Hassan Sadek, Suhail K. Siddique, Chien Chen, Rong-Ming Ho

**Affiliations:** † Department of Chemical Engineering, National Tsing Hua University, Hsinchu 30013, Taiwan

**Keywords:** bicontinuous nanohybrids, well-ordered nanostructure, block copolymer, templated synthesis, nanosize effect, hybridization effect, mechanical metamaterials

## Abstract

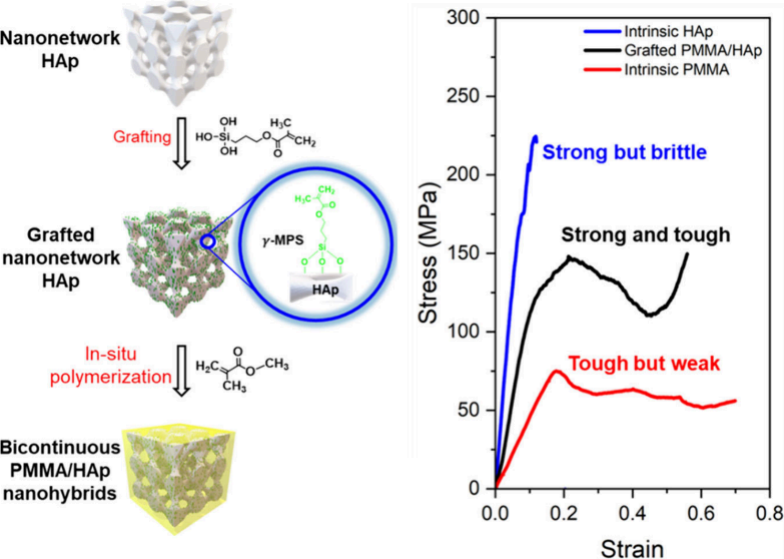

Biomimicking natural structures to create structural materials with superior mechanical performance is an area of extensive attention, yet achieving both high strength and toughness remains challenging. This study presents a novel bottom-up approach using self-assembled block copolymer templating to synthesize bicontinuous nanohybrids composed of well-ordered nanonetwork hydroxyapatite (HAp) embedded in poly(methyl methacrylate) (PMMA). This structuring transforms intrinsically brittle HAp into a ductile material, while hybridization with PMMA alleviates the strength reduction caused by porosity. The resultant bicontinuous PMMA/HAp nanohybrids, reinforced at the interface, exhibit high strength and toughness due to the combined effects of topology, nanosize, and hybridization. This work suggests a conceptual framework for fabricating flexible thin films with mechanical properties significantly surpassing those of traditional composites and top-down approaches.

Nature develops the ability to combine inorganic and organic materials with sophisticated structures resulting in hybrids with exceptional mechanical properties.^1−3^ Extensive studies have been performed to fabricate hybrid materials with bicontinuous textures, especially network-structured materials, giving a groundbreaking departure from conventional hybrid materials for superior mechanical performance.^4−8^ Incorporating dissimilar materials into hybrids can simultaneously enhance modulus, strength, toughness, impact resistance, thermal stability, and biocompatibility.^8−10^ Over the past decades, top-down approaches such as 3D printing and lithography have been used to expand the concept of micro/nanolattice (network-structured) materials for enhanced mechanical performance.^11−16^ Meanwhile, a straightforward approach enhances mechanical properties such as strength by reducing the flaws and defects, which can be obtained by reducing the size.^17,18^ However, the network structure at nanosize from a top-down approach remains challenging.

In contrast to the top-down approach, which is limited in the fabrication of sub-micrometer strut size, block copolymer (BCP) self-assembly provides a cost-effective approach for fabricating well-ordered network structures (e.g., gyroid and diamond) at the nanoscale.^19−21^ By utilizing self-assembled BCP for templated syntheses, a variety of well-ordered nanonetwork materials can be successfully fabricated.^22−27^ By removal of the polymer template, it is possible to fabricate gyroid- and diamond-structured inorganics. Inspired by knobby starfish, a well-ordered nanonetwork calcite single crystal (CSC) can be fabricated by a templated crystallization reaction using diamond-structured BCP, giving a brittle-to-ductile transition due to the topology effect. With the same morphology as knobby starfish but a smaller feature size, the nanonetwork CSC from the templated synthesis exhibits higher specific strength and larger energy absorption per volume.^28^ Although a significant enhancement in energy absorption was achieved due to the well-ordered nanonetwork structure obtained from BCP templated synthesis, porosity in materials presents a trade-off between mechanical performance and weight. Voids act like microcracks that decrease both strength and stiffness. Yet, the pores significantly reduce weight, giving high specific mechanical properties. For practical applications, it is necessary to consider this trade-off for attaining optimal mechanical performance. Nature overcomes this challenge by combining inorganic and organic materials to form hybrids, giving exceptional properties, as seen in mantis shrimp, nacre, wood, and bone. Interestingly, the dactyl club of mantis shrimp has impressive impact resistance capability, outperforming many engineered materials, due to a unique adaptation for withstanding high-speed collisions during their feeding activities.^29^ The impact surface (∼70 μm thick) of the dactyl club consists of bicontinuous nanonetwork hydroxyapatite embedded in an organic matrix, resulting in an extraordinary combination of stiffness and toughness.^30,31^ Such properties are mainly driven by the combined effects of the topology of the bicontinuous structure and hybridization.

Herein, this work aims to mimic the structural design motifs of the impact surface of the dactyl club of mantis shrimp through the fabrication of bicontinuous nanohybrids. By exploiting templated synthesis using self-assembled BCP with diamond structure as a template, it is possible to fabricate well-ordered nanonetwork ceramics that overcome the fabrication of feature size limitation from a top-down approach, achieving a brittle-to-ductile transition due to the deliberate structuring as a network in the nanoscale. Using nanonetwork ceramics as the subsequent template, the issue of the porosity-related reduction in modulus and strength in network materials can be addressed by templated polymerization of monomers for hybridization. Reinforcing interfacial strength in the fabricated nanohybrids allows for the simultaneous achievement of high strength and toughness due to the combined effects of topology, nanosize, and hybridization.

## Fabrication Strategy and Characterization

**Figure 1:**
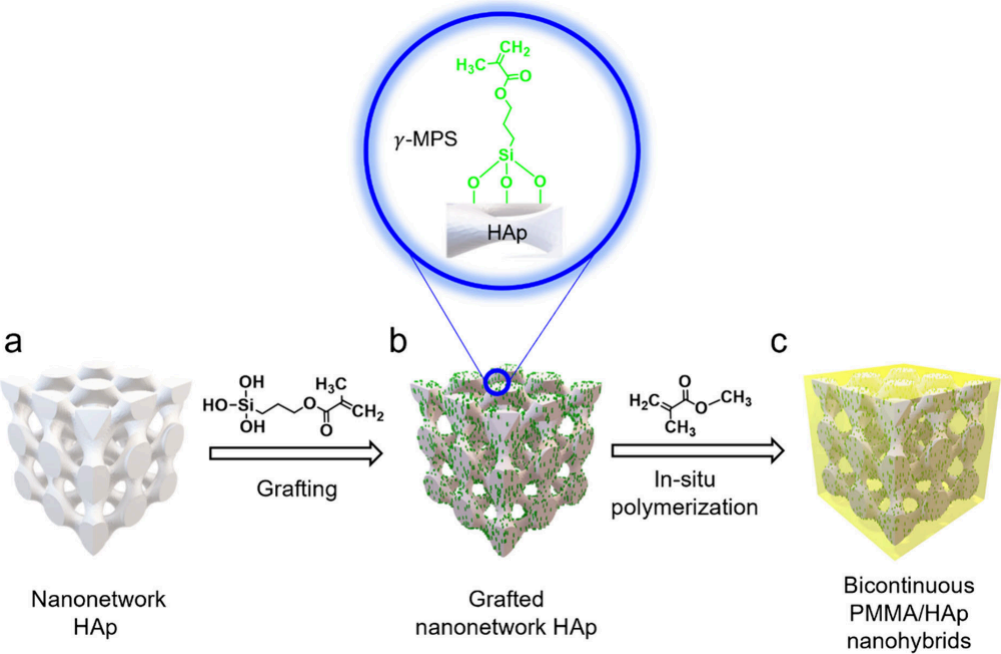
Schematic illustration for design and fabrication procedures of bicontinuous PMMA/HAp nanohybrids. (a) Nanonetwork HAp fabricated by templated sol–gel reaction using nanoporous PS fabricated from self-assembled PS-*b*-PDMS as a template. (b) Nanonetwork HAp grafted with γ-MPS to functionalize the HAp with reactive methacrylate. (c) Well-ordered bicontinuous PMMA/HAp nanohybrids obtained by templated polymerization of MMA into PMMA using (b) as a subsequent template.

Figure 1 illustrates the fabrication procedures of the targeted bicontinuous poly(methyl methacrylate)/hydroxyapatite (PMMA/HAp) nanohybrids with diamond-structured HAp embedded in the PMMA matrix, which mimics the structural features of the dactyl club of mantis shrimp with nanonetwork HAp embedded in chitin and/or protein. By using polystyrene-*block*-polydimethylsiloxane (PS-*b*-PDMS) from controlled self-assembly followed by hydrofluoric acid (HF) etching of PDMS in a PS matrix, nanoporous PS with well-ordered nanochannels can be fabricated and used as a template.^32^ Fabrication of well-ordered nanonetwork HAp with a diamond structure (Figure 1a) can be achieved by templated sol–gel reaction of calcium nitrate and triethyl phosphite (Figure S1);^33^ the diamond-structured HAp is referred to as a hard component for the targeted nanohybrids. For hybridization, PMMA is used as a soft component for nanohybrids. For the fabrication of the aimed nanohybrids, methyl methacrylate monomer was polymerized using diamond-structured HAp as a template. As is known for all heterophase hybrids, it is crucial to create strong interfacial strength between organic and inorganic components, giving nanohybrids superior mechanical performance. To acquire strong bonding at the interface, the nanonetwork HAp will be chemically grafted with 3-(trimethoxysilyl)propyl methacrylate (γ-MPS) to implant a methacrylate group as a reactive site on the surface of the nanonetwork HAp (Figure 1b), giving improvement of the affinity of the HAp and PMMA. Consequently, bicontinuous PMMA/HAp nanohybrids (Figure 1c) can be fabricated with strong interfacial strength to ensure the structural continuity for the nanohybrids fabricated with the aimed mechanical performance.

**Figure 2:**
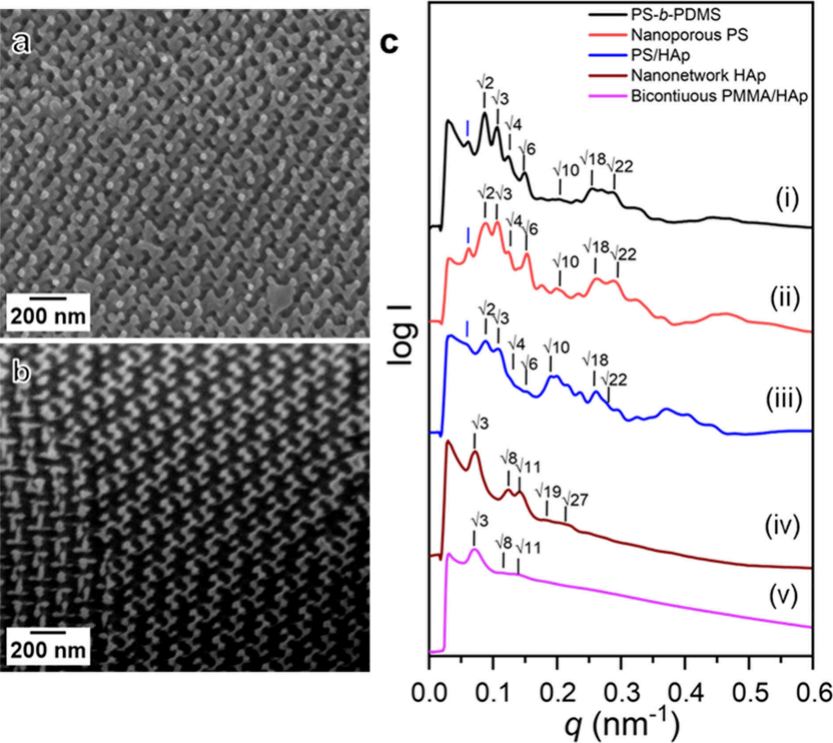
Well-ordered bicontinuous PMMA/HAp nanohybrids fabricated by templated syntheses using nanoporous PS fabricated from self-assembled PS-*b*-PDMS as a first template followed by the use of nanonetwork HAp as a subsequent template. (a) FESEM micrograph of a HAp nanonetwork before PMMA infiltration. (b) Cross-sectional FESEM micrograph of bicontinuous PMMA/HAp nanohybrids. (c) 1D SAXS profiles of (i) self-assembled PS-*b*-PDMS, (ii) fabricated nanoporous PS template, (iii) PS/HAp before removal of the PS template, (iv) HAp nanonetwork, and (v) bicontinuous PMMA/HAp nanohybrids.

Figure 2a shows the FESEM image of the HAp nanonetwork with diamond textures that was fabricated after multistage thermal treatment, giving the calcination of HAp with high crystallinity and removal of the PS template while preserving the aimed morphology.^33^ Note that the HAp nanonetwork features well-ordered microdomains, each with a strut size of approximately 20 nm and a grain size of approximately 500 nm in diameter (Figure S2). To validate the grafting of the HAp nanonetwork with γ-MPS, X-ray photoelectron spectroscopy (XPS) was employed; an additional peak in the Si 2p region can be found to evidence the grafting of γ-MPS on the surface of the HAp nanonetwork (Figure S3). Subsequently, focused ion beam (FIB) milling was used to observe the internal morphology of the bicontinuous PMMA/HAp nanohybrids after the infiltration of PMMA by templated polymerization of the MMA monomer using the grafted HAp nanonetwork as a template. As shown in Figure 2b, based on SEM observation with backscattered electron imaging (BEI) mode, a typical diamond pattern with bright HAp in dark PMMA evidenced the formation of the PMMA/HAp nanohybrids. Moreover, elemental mapping using energy-dispersive X-ray spectroscopy (EDS) on the bicontinuous PMMA/HAp nanohybrids (Figure S4) exhibits a uniform distribution of calcium, phosphorus, oxygen, carbon, and silicon, further evidencing the success of the grafting process. To further confirm the observed morphologies, small-angle X-ray scattering (SAXS) was used to examine the fabricated materials. The 1D SAXS profile of PS-*b*-PDMS gives reflections at the relative *q* values of √2, √3, √4, √6, √10, √18, and √22, in line with the predicted reflections from the double-diamond (DD) structure (Figure 2c(i)). Note that the weak peak at the low-*q* region (blue line) is attributed to a slight deformation of the forming DD structure from self-assembly. After removal of PDMS, the 1D SAXS profile of nanoporous PS shows no significant change in the relative *q* values (Figure 2c(ii)), confirming the successful fabrication of the PS template with well-ordered nanochannels. The 1D SAXS profile of the fabricated PS/HAp exhibits scattering results with reflections at similar relative *q* values (Figure 2c(iii)). After the removal of the PS template, the 1D SAXS profile of the diamond-structured HAp shows reflections occurred at the relative *q* values of √3, √8, √11, √19, and √27 (Figure 2c(iv)); this is attributed to the shifting of two diamond structures, giving single diamond-like reflections.^34^ Note that the shrinkage of the network structure after calcination is attributed to the densification of the HAp. The scattering results are in line with the morphological observation (see Figure S5). After the templated polymerization of MMA with the use of the nanonetwork HAp as a subsequent template, the 1D SAXS of PMMA/HAp nanohybrids resembles the profile of the HAp nanonetwork but with lower density for higher-order reflections (Figure 2c(v)), implying that the successful templated polymerization can be achieved to give a lower electron density contrast for scattering, resulting in the reduction in scattering intensity. As a result, the platform for the fabrication of well-ordered nanohybrids can give a facile approach for the target materials to give the aimed PMMA/HAp nanohybrids with well-defined nanonetwork HAp embedded in PMMA. Note that conventional nanohybrids obtained with a dispersion of inorganic nanoparticles in an organic matrix suffer from the aggregation and discontinuity of inorganic reinforcing materials, causing an inhomogeneous distribution and thus reduction in mechanical performance.^35,36^ By contrast, the well-ordered bicontinuous nanohybrids fabricated offer uniform dispersion and percolation properties; this is expected to enhance load transfer efficiency and impact resistance through a homogeneous stress distribution along continuous struts.

## Mechanical Performance for Fabricated Bicontinuous PMMA/HAp Nanohybrids

**Figure 3:**
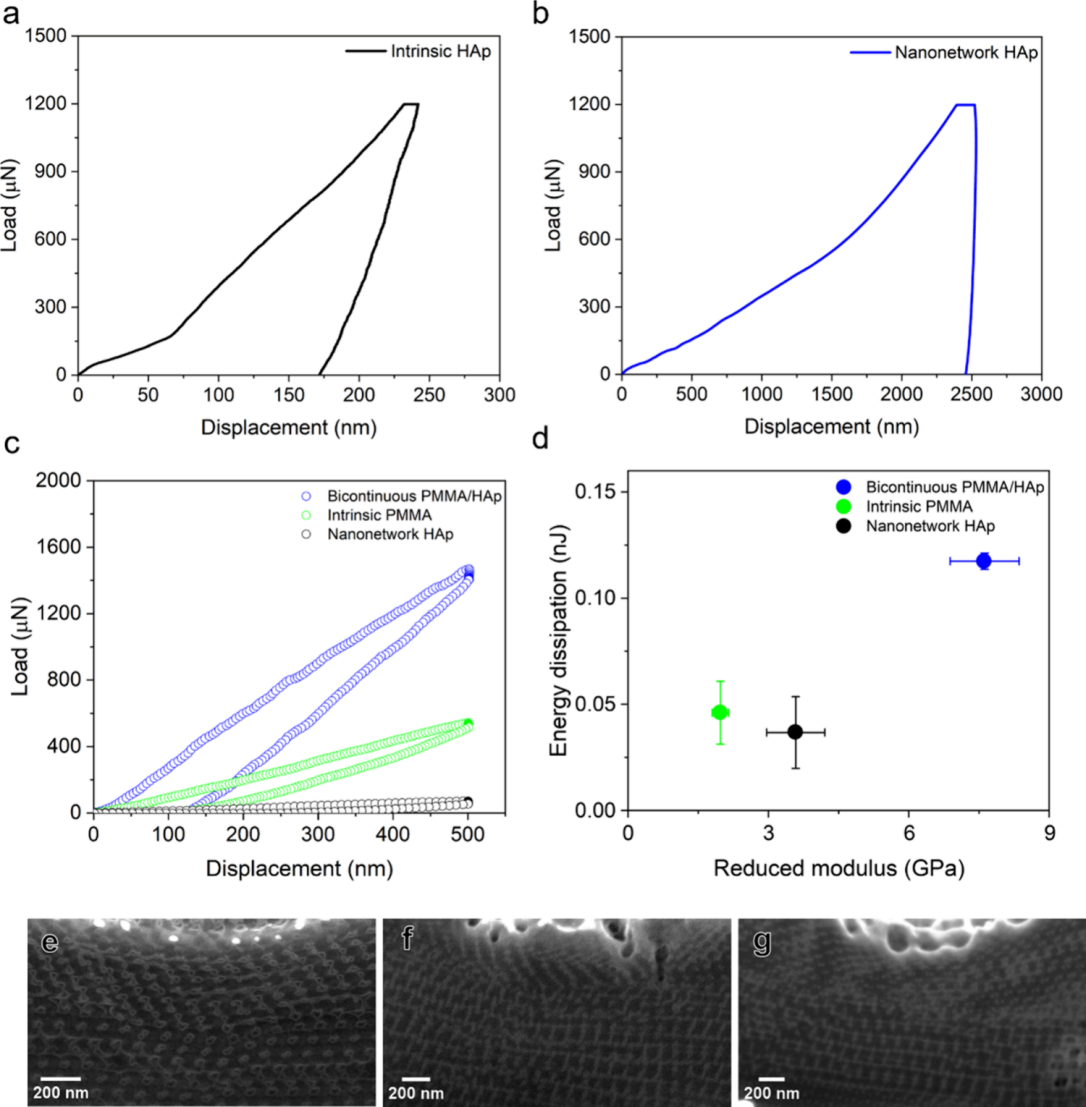
Topology and hybridization effects on mechanical properties of bicontinuous PMMA/HAp nanohybrids. Load–displacement curves for nanoindentation tests of (a) intrinsic HAp and (b) nanonetwork HAp at a constant load of 1200 μN. (c) Load–displacement of PMMA/HAp nanohybrids, intrinsic PMMA, and HAp nanonetwork at constant displacement. (d) The plot of energy dissipation versus reduced modulus for nanonetwork HAp, intrinsic PMMA, and the PMMA/nanohybrids. Postindentation FESEM micrographs corresponding to loads of (e) 400, (f) 800, and (g) 1200 μN.

To explore the impact of the nanonetwork structure on the mechanical characteristics of fabricated HAp, a nanoindentation test was conducted on the nanonetwork HAp in comparison with intrinsic HAp. In contrast to the intrinsic brittle HAp (Figure 3a), the load–displacement curve of the nanonetwork HAp exhibits a graceful deformation pattern without cracking events (Figure 3b). Notably, the nanonetwork HAp displays significant permanent displacement (i.e., plastic deformation) more than 15 times the intrinsic HAp at a maximum load of 1200 μN. This observation features the substantial effect of deliberate structuring as nanonetwork texture on the mechanical properties of HAp, leading to a brittle-to-ductile transition and thus an enhanced capacity for energy dissipation. Note that the assessment of energy dissipation capacity involves the integration of the area beneath the load–displacement curve. By calculating the integrated area at a maximum loading of 1200 μN, the nanonetwork HAp exhibits a substantial increase, measuring 1.25 nJ as compared to the intrinsic HAp, with a value of 0.11 nJ.

Figure 3c shows the load–displacement curves of intrinsic PMMA, the nanonetwork HAp, and the bicontinuous PMMA/HAp nanohybrids for systematic comparisons. As previously noted, the porosity of the nanonetwork HAp affects the mechanical performance, leading to a reduction of modulus and strength. Therefore, PMMA infiltration is expected to alleviate the reduction in mechanical performance. Unlike constant loading, nanoindentation tests were conducted at constant displacement, ensuring direct comparison without the influence of the varying indentation depths. This approach minimizes the material variability by maintaining a consistent indentation depth, enhancing the reliability of the comparison. For the nanonetwork HAp, a 500 nm displacement can be achieved with low loading force, consistent with nanoindentation under constant loading. After hybridization with the PMMA, the PMMA/HAp nanohybrids demonstrate a significant increase in loading capacity to 1500 μN at 500 nm displacement, three times higher than that of intrinsic PMMA. The combination of hybridization and deliberate structuring effectively addresses the porosity issue, improving the modulus and strength of the PMMA/HAp nanohybrids compared to the nanonetwork HAp and intrinsic PMMA. The reduced modulus of the PMMA/HAp nanohybrids is 7.6 ± 0.7 GPa, approximately double that of nanonetwork HAp (3.5 ± 0.6 GPa) and four times higher than intrinsic PMMA (1.9 ± 0.17 GPa). Additionally, the energy dissipation capability of the PMMA/HAp nanohybrids, as shown in Figure 3d, is significantly enhanced. The integration area of the closed loop of the load–displacement curve measures 0.12 nJ, about three times higher than that of nanonetwork HAp (0.04 nJ) and two times higher than that of intrinsic PMMA (0.05 nJ). It is reasonable to expect that the PMMA/HAp nanohybrids experience the typical deformation mechanism as ductile materials. To further investigate the deformation mechanism of the PMMA/HAp nanohybrids, postindentation FESEM images of residual indented locations at maximum loads of 400, 800, and 1200 μN were analyzed. The initial deformation of the HAp/PMMA nanohybrids at a loading of 400 μN suggests primary distortion through bending of the nanonetwork HAp struts, allowing stress distribution without cracking, consistent with the nanoindentation results (Figure 3e). Subsequent strut bending results in extensive plastic deformation (Figure 3f), characteristic of ductile materials, resulting from the deliberate structuring of the HAp nanonetwork combined with the ductile nature of PMMA. As the load increases to 1200 μN, the absence of discontinuities and crack propagation near the contact area (Figure 3g) indicates complete distortion of PMMA/HAp due to plastic deformation. This demonstrates projected ductility, highlighting bending-dominated behaviors under loading for enhanced energy dissipation.^9^ Consequently, the mechanical behavior of the PMMA/HAp nanohybrids is underpinned by the synergistically combined effects of hybridization and deliberate structuring, incorporating hard nanonetwork HAp within the soft PMMA matrix.

**Figure 4:**
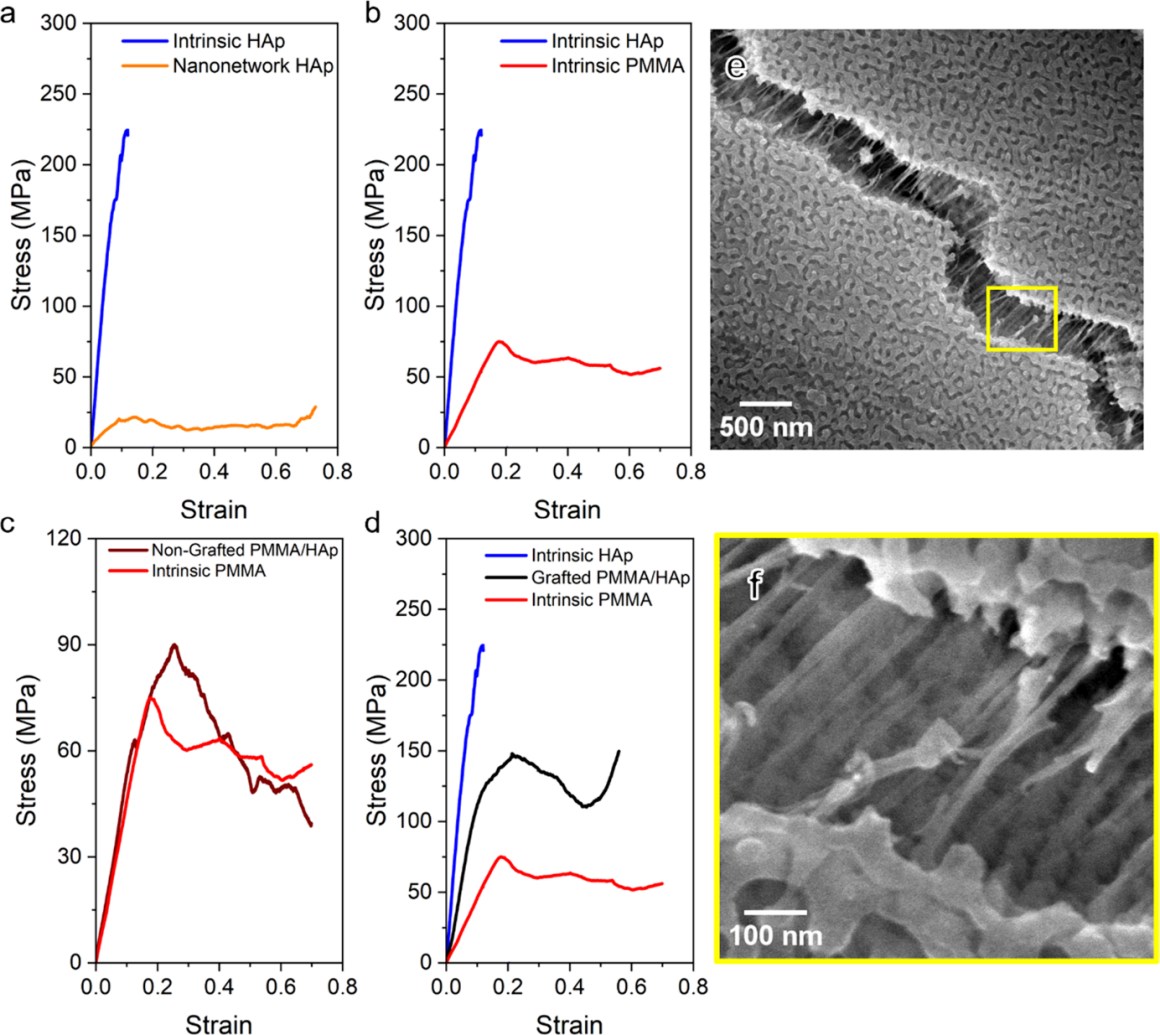
Mechanical performance of bicontinuous PMMA/HAp nanohybrids. Engineering stress–strain curve of (a) intrinsic HAp versus nanonetwork HAp. (b) Intrinsic HAp versus intrinsic PMMA. (c) Intrinsic PMMA versus nongrafted PMMA/HAp nanohybrids. (d) Grafted PMMA/HAp nanohybrids as compared to intrinsic HAp and intrinsic PMMA. (e) Low-magnified FESEM image of the fractured surface in grafted PMMA/HAp nanohybrids. (f) Magnified SEM image taken from the yellow boxed area in (e).

To further examine the effect of hybridization on the mechanical properties of PMMA/HAp nanohybrids, pillar-shaped HAp/PMMA nanohybrids with a diameter of approximately 2 μm were obtained by FIB milling. Figure 4 exhibits representative engineering stress–strain curves for the PMMA/HAp nanohybrids with and without grafting for systematic comparison with intrinsic HAp and nanonetwork HAp. In the stress–strain curve for intrinsic HAp (Figure 4a), a notable strain burst occurs after compression, characteristic of brittle materials displaying initial linear elasticity with a calculated Young’s modulus of 2.6 GPa. Microcrack propagation causes a discontinuity in the curve, leading to catastrophic failure at a compression strength of approximately 227 MPa, with no evident plastic deformation. Conversely, nanonetwork HAp demonstrates a prolonged plateau with gradual layer-by-layer collapse, six times higher than that of intrinsic HAp due to intentional nanonetwork structuring. Despite the enhanced energy absorption, there was a substantial reduction in Young’s modulus (0.255 GPa) and strength (23 MPa), caused by the porosity effect on mechanical performance. By contrast, the intrinsic PMMA shows a large plateau after reaching compressive strength at approximately 75 MPa (Figure 4b), as expected from the polymeric material. The stress–strain curve of the PMMA/HAp nanohybrids shows an enhancement modulus (0.53 GPa) and strength (89 MPa) as compared to the PMMA matrix (Figure 4c). Yet, there is a small strain burst in the curve that might be caused by insufficient pore filling and/or the poor interfacial bonding between HAp and PMMA, giving inhomogeneous deformation (see Figure S6). Most importantly, there is no significant variation for the nanohybrids fabricated as compared to the intrinsic PMMA; we speculate that the insignificance is mainly caused by poor interfacial bonding. To address the problem of weak interfacial strength, the surface of the nanonetwork HAp was grafted with γ-MPS to enhance bonding. The stress–strain curve of the PMMA/HAp nanohybrids with the grafting treatment shows a significant enhancement in mechanical performance (Figure 4d). After the grafting treatment, there is no noticeable strain burst because of homogeneous deformation, evidencing the stronger bonding between HAp and PMMA. Surprisingly, these nanohybrids demonstrate a superior Young’s modulus of 1.2 GPa, exceeding intrinsic PMMA (0.4 GPa) and closely approaching intrinsic HAp (2.6 GPa). Moreover, the strength of the PMMA/HAp nanohybrids reaches approximately 120 MPa before experiencing a plateau with a minor decline and finally reaching a peak stress of approximately 148 MPa, resulting in high energy absorption per volume of 172.1 MJ/m^3^ at a given strain magnitude in comparison with the intrinsic HAp (9.5 MJ/m^3^) and nanonetwork HAp (13.8 MJ/m^3^) as well as the nanohybrids without grafting treatment (98 MJ/m^3^). Those results demonstrate the feasibility of effectively solving the porosity problem in mechanical performance for the nanonetwork HAp by backfilling nanonetwork HAp with soft PMMA, giving nanohybrids with high elastic modulus, high compression strength, and superior energy absorption capability combining the characteristics of hard and soft materials.

To further identify the key factors contributing to the enhanced mechanical performance of the bicontinuous PMMA/HAp nanohybrids, a crack propagation on the fractured surface was visualized by FESEM. As shown in Figure 4e,f, the crack displays clear evidence of polymer tearing and stretching over micrometer dimensions to prevent crack propagation^2,37^ with the formation of a fibrillar texture that further evidences the strong interfacial strength between the PMMA and HAp, suggesting that the PMMA intrinsically provides an additional mechanism for the enhancement of energy absorption, while the nanonetwork HAp gives rise to the deliberate structuring effect on the energy dissipation capability (see Figure S7). This method enables us to precisely observe how damage mechanisms develop ahead of the crack and the subsequent external strengthening mechanisms. Additionally, this allows us to continuously capture how deliberate structuring influences these mechanisms. These findings reveal a convergence of strengthening mechanisms operating at various size scales, similar to what has been observed in natural biological materials.^38^

**Figure 5:**
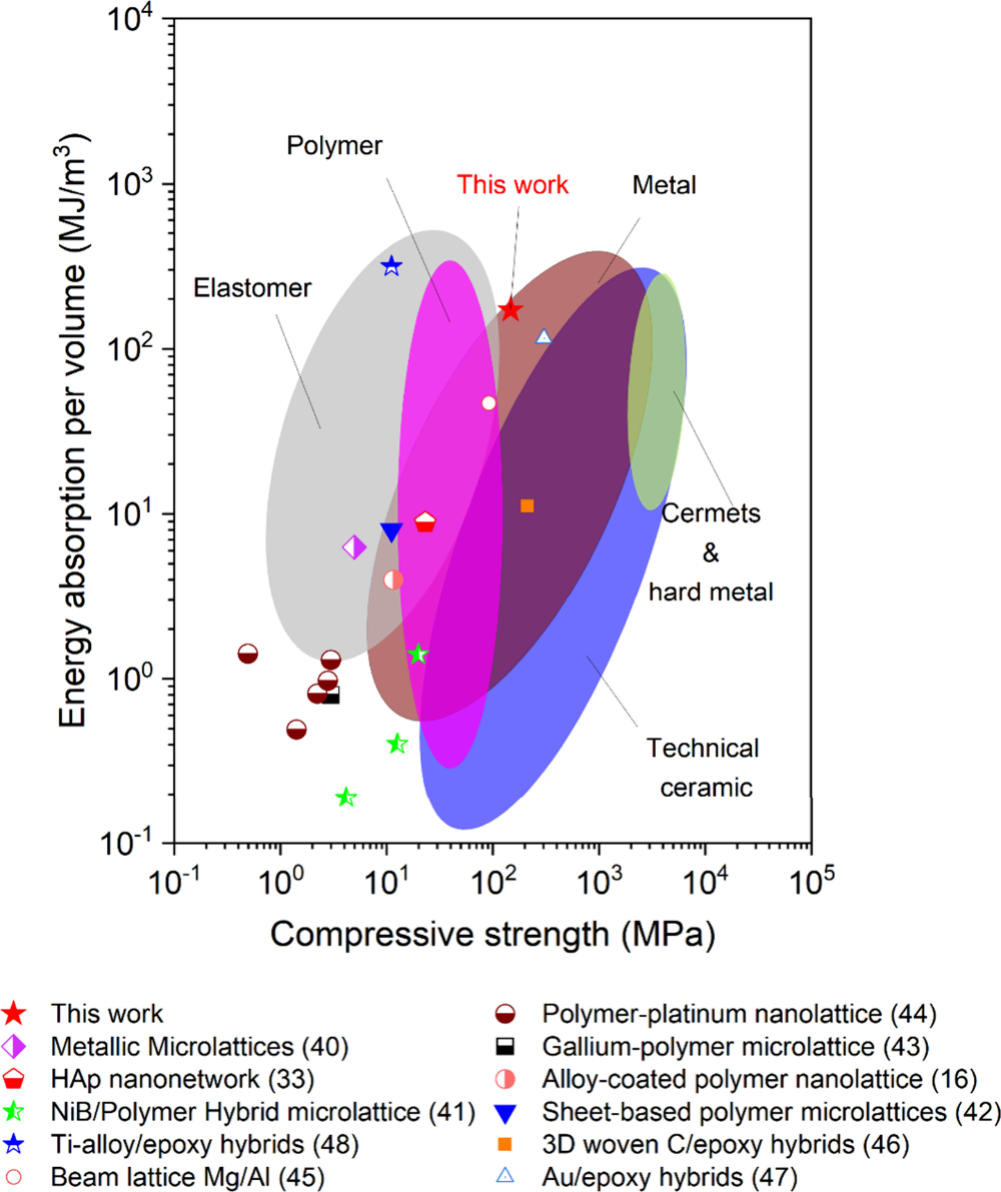
Comparison of mechanical properties. Ashby plot of compressive strength as a function of energy absorption per volume for the comparison of the bicontinuous PMMA/HAp nanohybrids and micro/nanolattice materials previously reported as well as structured materials under deformation with equivalent strain.

To evaluate the mechanical capabilities of the PMMA/HAp nanohybrids, it is crucial to compare them with various materials, thereby gaining a comprehensive understanding of their strengths and energy absorption. This comparative analysis extends beyond traditional bulk materials to include micro/nanolattice structures and other hybrids formed through top-down approaches.^16,39−44^ By assessing these materials through uniaxial compression at a 50% strain level, we gave insights into their performance and suitability for demanding applications. Figure 5 shows the Ashby plot of compressive strength as a function of energy absorption per volume for the PMMA/HAp nanohybrids fabricated in this study and structured materials as well as different micro/nanolattice materials previously reported, giving the systematic comparison of the mechanical performance of the PMMA/HAp nanohybrids fabricated to their counterparts. The PMMA/HAp nanohybrids, characterized by their intricate structure, lie comfortably within the high strength and high energy absorption efficiency spectrum. Due to the deliberate structuring of the well-ordered bicontinuous nanohybrids, the PMMA/HAp nanohybrids possess the dual characteristics of organics and inorganics, achieving a superior balance in mechanical performance. As a result, the PMMA/HAp nanohybrids not only outperform conventional bulk materials but also surpass advanced materials like cermets and hard metals, which are renowned for their exceptional mechanical properties. This exceptional performance is indicative of the nanohybrids surpassing that of previously reported studies on interpenetrating phases, typically composed of lightweight matrix materials like epoxy.^45−47^ What sets the PMMA/HAp nanohybrids apart is the magnitude of their superiority, often exceeding that of interpenetrating phases by more than 1 order of magnitude. This remarkable achievement solidifies their status as advanced materials specifically tailored for high-performance lightweight applications.

## Discussion

In conclusion, this study presents a successful method for replicating the structural characteristics present in the impact surface of mantis shrimp. By utilizing BCP templating synthesis, nanohybrids with nanonetwork structures, incorporating HAp and PMMA can be fabricated. Robust interfacial bonding, enhanced by chemical grafting, is crucial for optimizing the modulus, strength, and energy absorption. Nanoindentation and microcompression tests reveal that the resulting bicontinuous PMMA/HAp nanohybrids display a marked improvement in mechanical performance compared to intrinsic HAp, intrinsic PMMA, and nanonetwork HAp. The grafting process significantly enhances pore filling and strengthens the interfacial bonding between the organic and inorganic phases. This study suggests significant potential for developing innovative materials with superior mechanical properties, surpassing those of conventional hybrids and micro/nanolattice materials through a combination of deliberate structuring, hybridization, and nanoscale effects.

## Supporting Information Available

Materials and methods, synthesis of diamond-structured PS template, fabrication of nanonetwork HAp (Figure S1), mechanism underlying the deformation of PMMA/HAp nanohybrids under nanoindentation test, characterization of diamond-structured HAp (Figure S2), characterization of grafted HAp nanonetwork (Figure S3), EDX images of bicontinuous PMMA/HAp nanohybrids (Figure S4), TEM images of PS-*b*-PDMS (Figure S5), influence of interfacial bonding on mechanical performance and pore-filling efficiency (Figure S6), postindentation FESEM micrograph of the underlying surfaces exposing the contact deformation of grafted PMMA/HAp nanohybrids (Figure S7) (PDF)
