# Supplementary material for: Well-Ordered Bicontinuous Nanohybrids from a Bottom-Up Approach for Enhanced Strength and Toughness
Source: Nano Lett. 2024 Aug 28;24(35):11020–7. doi: 10.1021/acs.nanolett.4c03157 (PMC11378333; doi:10.1021/acs.nanolett.4c03157)
Supplement: Supplementary file 1 — nl4c03157_si_001.pdf [file nl4c03157_si_001.pdf]

# Well-Ordered Bicontinuous Nanohybrids from Bottom-Up Approach for Enhanced Strength and Toughness

*Hassan Sadek[1], Suhail K. Siddique[1], Chien Chen, Rong-Ming Ho[1*]*

Department of Chemical Engineering, National Tsing Hua University, Hsinchu 30013, Taiwan

E-mail:rmho@mx.nthu.edu.tw

**This PDF file includes:**

Materials and Methods
Supplementary Text
Figures. S1 to S7
References

## Materials and Characterization

Calcium nitrate tetrahydrate ($Ca(NO_3)_2 \cdot 4H_2O$, 99%, Sigma-Aldrich), triethyl phosphite ($(C_2H_5O)_3P$, Matrix Scientific), polyvinylpyrrolidone (PVP, Mw~3,500 g/mol, Acros organic), calcium chloride dihydrate ($CaCl_2 \cdot 2H_2O$, 99%, Sigma-Aldrich), ammonium hydroxide ($NH_4OH$, 33-35 wt%, Honeywell), hydrofluoric acid (HF, Sigma-Aldrich), methyl methacrylate (MMA, Alfa Aesar), 3-(trimethoxysilyl)propyl methacrylate ($\gamma$-MPS, Sigma-Aldrich), 2,2-azobisisobutyronitrile (AIBN, Biotech), chloroform (J.T. Baker), ethanol (absolute, Honeywell), All chemicals were of analytical grade, and used as received without further purification.

## Methods

**Synthesis of PS-*b*-PDMS:** the synthesis process of lamellae-forming PS-*b*-PDMS was performed through anionic polymerization as described in our previous report.[1,2] Specifically, the molar masses of the constituent polymers, PS and PDMS, were determined to be 51,000 g/mol and 35,000 g/mol, respectively. This meticulous control over the molecular weights facilitated the fabrication of PS-*b*-PDMS characterized by an overall molar mass of 86,000 g/mol with the volume fraction of PDMS block of 0.42 in the PS-*b*-PDMS.

**Synthesis of diamond-structured PS-*b*-PDMS thin film:** The fabrication process was reported in our previous study.[3] Briefly, PS-*b*-PDMS (0.1g) synthesized above was dissolved in chloroform with a concentration of 10wt% in a 1ml vial and transferred to a square-grooved glass container. The film formation was accomplished by immersing a silicon wafer vertically into the solution, followed by drying at room temperature. Subsequently, a solvent annealing process was employed to regulate self-assembly, facilitating the development of a diamond-structured PS-*b*-PDMS thin film.

**Synthesis of diamond-structured PS template:** the fabrication process involved floating the diamond-structured PS-*b*-PDMS thin film in hydrofluoric acid (HF) solution (HF/methanol = 3/1 by volume) for 24 hr to selectively etch out the PDMS block. Afterward, the template underwent multiple washes with methanol to eliminate any residual HF solution, resulting in a nanoporous PS template with precisely defined nanochannels.

**Fabrication of nanonetwork HAp:** The templated sol-gel reaction was employed for the synthesis of nanonetwork HAp as reported in our previous work.[4] In this process, $Ca(NO_3)_2{\cdot}4H_2O$ and $(C_2H_5O)_3P$ were served as the precursors. Initially, a solution of 1M $Ca(NO_3)_2.4H_2O$ in 5ml ethanol and a stoichiometric amount of $(C_2H_5O)_3P$ with Ca/P of 1.67 in ethanol/$H_2O$ solvent with a molar ratio of $H_2O/P = 8$ were separately prepared and allowed to undergo hydrolysis at room temperature for one day with continuous stirring. Subsequently, the prepared solutions were mixed and stirred for thirty minutes at which the pH was adjusted to be approximately 8 using ammonium hydroxide. Polyvinylpyrrolidone (PVP) was introduced to control the growth of HAp particles. The nanoporous PS template was immersed in the solution, aged at 50 ºC for several days, and then collected on a substrate followed by drying at 70 ºC until a white gel was obtained. The dried gel underwent controlled multistage thermal treatments, giving well-ordered nanonetwork HAp in the thin-film state as a monolith.

**Fabrication of Bicontinuous PMMA/HAp Nanohybrids:** The nanonetwork HAp obtained from templated sol-gel synthesis, as described above was chemically grafted with 3-(trimethoxysilyl)propyl methacrylate (*γ*-MPS) by immersing the nanonetwork HAp in 10 wt% γ-MPS/ethanol solution for 24 h at ambient conditions and then dried at 80 °C for 2 h; this grafting process provides a methacrylate group on the surface of nanonetwork HAp to further react with MMA monomer during the infiltration with PMMA for enhancing the interface strength. In the

first step of PMMA polymerization, methyl methacrylate (MMA) undergoes free radical polymerization initiated by 2,2′-Azobisisobutyronitrile (AIBN) in a methanol solvent, using a relatively high initiator concentration (1 wt.% AIBN) for 1 hour at 70 °C. This initial reaction aims to maximize the formation of radicals on *γ*-MPS molecules attached to the surface of nanonetwork HAp, resulting in the chemical grafting of PMMA chains. This is followed by a second free radical polymerization process that is performed in bulk but with a lower initiator concentration (0.5 wt.% AIBN). This step was carried out for 3 days at 40 °C to ensure thorough infiltration of nanonetwork HAp. Finally, the bicontinuous PMMA/HAp nanohybrid was heated at 90 °C for 2 h to guarantee complete polymerization. Non-grafted PMMA/HAp nanohybrids were fabricated using only the final polymerization step.

**Material characterization:** The diamond-structured configurations of PS-*b*-PDMS, PS/HAp, and HAp were subjected to characterization through transmission electron microscopy (TEM) utilizing a JEOL JEM-2100 LaB6 operating at an accelerating voltage of 200 kV. Sample preparation involved initial sectioning using a Leica UC6 Ultramicrotome, resulting in micro-sections with an approximate thickness of 70 nm. Field-emission scanning electron microscopy (FESEM) images were acquired using a HITACHI SU8010, and the elemental composition of the surfaces was investigated through energy-dispersive spectroscopy. Small-angle X-ray scattering (SAXS) experiments were conducted at beamline TLS-23A and TPS-13A, employing an X-ray beam with a diameter of 0.5 mm and energy of 10 keV (wavelength = 1.24 Å). 1D SAXS profiles were generated by integrating a 2D SAXS pattern collected using a MAR CCD X-ray detector (Dectris EIGER X 9M and Dectris EIGER X 1M). Wide-angle X-ray diffraction (WAXD) analysis was carried out using a Rigaku Dmax 2200 X-ray diffractometer with Cu Kα radiation, spanning an angle range from 10° to 80° at a scan rate of 1° $min^{-1}$.

**Nanoindentation Test:** Nanoindentation testing was conducted under ambient conditions utilizing the Hysitron Triboindenter equipped with a blunt (cono-spherical) tip with a 1 μm radius employing a continuous depth-sensing indentation technique. Maximum loads were set at 400, 800, and 1200 μN, with a loading/unloading rate and holding time of 60 μN/sec and 5 sec, respectively.

**Microcompression Test:** Preceding the microcompression test, pillar-shaped PMMA/HAp nanohybrids, intrinsic HAp, and intrinsic PMMA, each with an approximate diameter of 2 μm, were fabricated using focused ion beam (FIB) milling (Helios Nano Lab 600, FEI). The milling process involved an accelerating voltage of 30 kV with a beam current of 0.44 nA for the initial milling of the surrounding area, followed by final cutting with a beam current of 9.7 pA to mitigate ion beam-induced damage. Uniaxial compression tests were performed using a Hysitron Triboindenter equipped with a flat indenter (tip radius of approximately 5 μm) at a loading rate of 10 nm $s^{-1}$. The pillar of each sample was compressed until densification to evaluate the maximum energy absorption.

**Mechanism underlying the deformation of PMMA/HAp nanohybrids under nanoindentation test:** To understand the mechanism underlying the mechanical deformation of PMMA/HAp nanohybrids, cross-section FESEM of the executed area after nanoindentation test using blunt contact (spherical indenter) was closely observed (**Figure S7a**). As observed from the high magnification micrographs, differences between the executed region located directly beneath the indenter in comparison with the surrounding region can be identified. As shown in **Figure S7b**, nanonetwork HAp (white domain) reveals significant nanonetwork densification integrated within the PMMA matrix (dark domain), which gives an additional toughening mechanism. Meanwhile, the morphological observation in the executed region surrounding the contact damage remains

preserved without the occurrence of crack propagation (**Figure S7c**). Note that HAp is known for its exceptional hardness and brittleness, likely initiating stress concentration around the indenter tip, which can lead to crack propagation due to the intense stress in that region. The morphological result after indentation indicates the nanonetwork densification of HAp plays a significant role in absorbing the impact energy. Meanwhile, the flexible and ductile nature of the PMMA matrix gives rise to plastic deformation, thus effectively supporting the brittle HAp without catastrophic mechanical failure. This dynamic interaction between the HAp and PMMA components due to the strong interfacial bonding enables the PMMA/HAp nanohybrids to distribute applied load, minimizing crack propagation, and thus giving rise to superior mechanical performance. This intricate interplay of materials within the nanohybrids during nanoindentation highlights the importance of understanding the synergistic effects of deliberate structuring and hybridization in designing advanced materials for various applications.

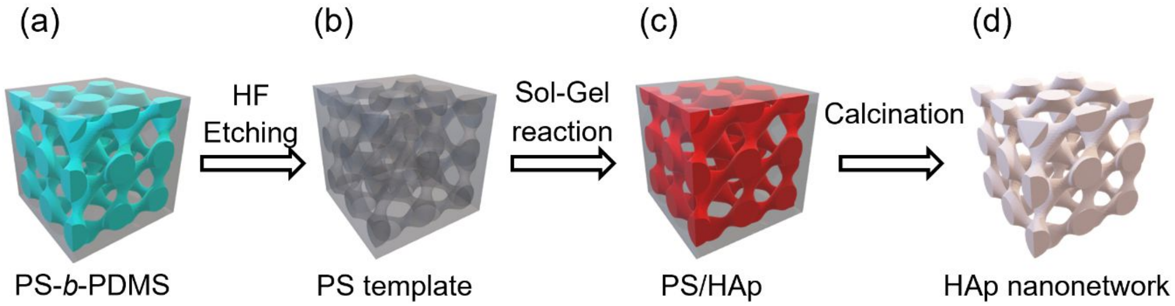


**Figure S1. Schematic illustration of the fabrication of HAp nanonetwork.** (a) PS-*b*-PDMS with diamond structure fabricated by solution casting of lamellae-forming PS-*b*-PDMS using chloroform as a solvent selective to PS. (b) PS template with nanopores featuring precisely defined nanochannels obtained by etching PDMS blocks in PS-*b*-PDMS using HF. (c) By exploiting the templated sol-gel reaction, the HAp can be fabricated inside the nanochannels of the PS template giving PS/HAp nanohybrids. (d) HAp nanonetwork resulting from thermal treatment of PS/HAp nanohybrids for removal of PS template.

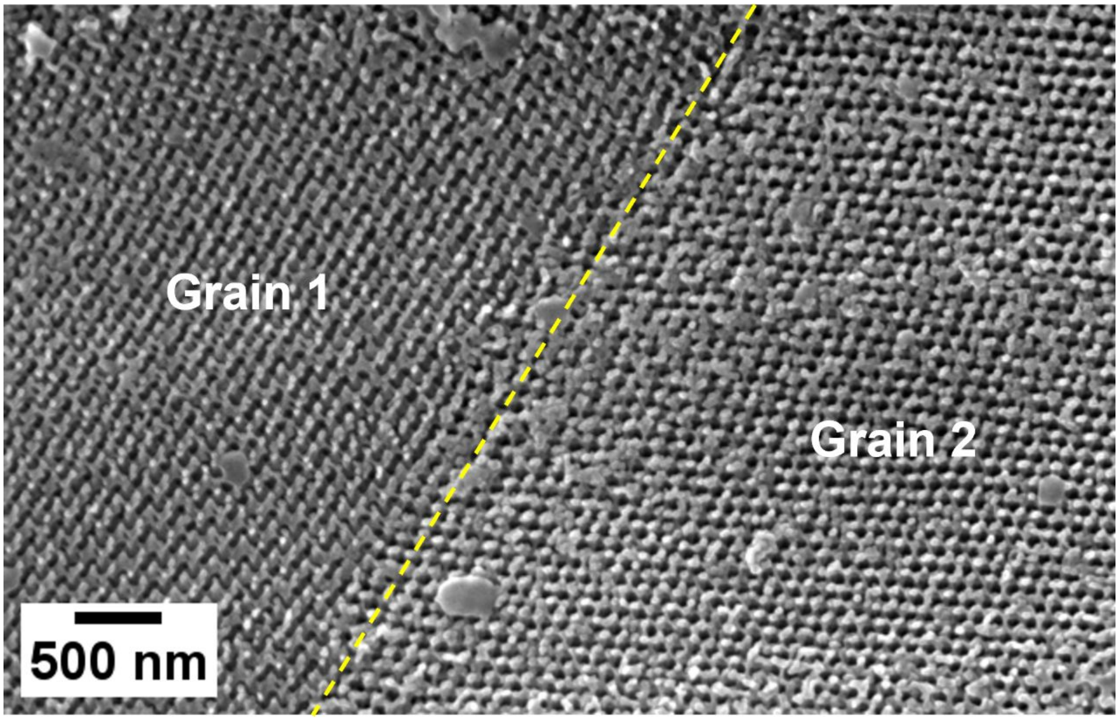


**Figure S2. Diamond-structured HAp**. FESEM micrograph of SEM micrograph of nanonetwork HAp after removal of the PS template by calcination, showing a grain boundary between two diamond domains.

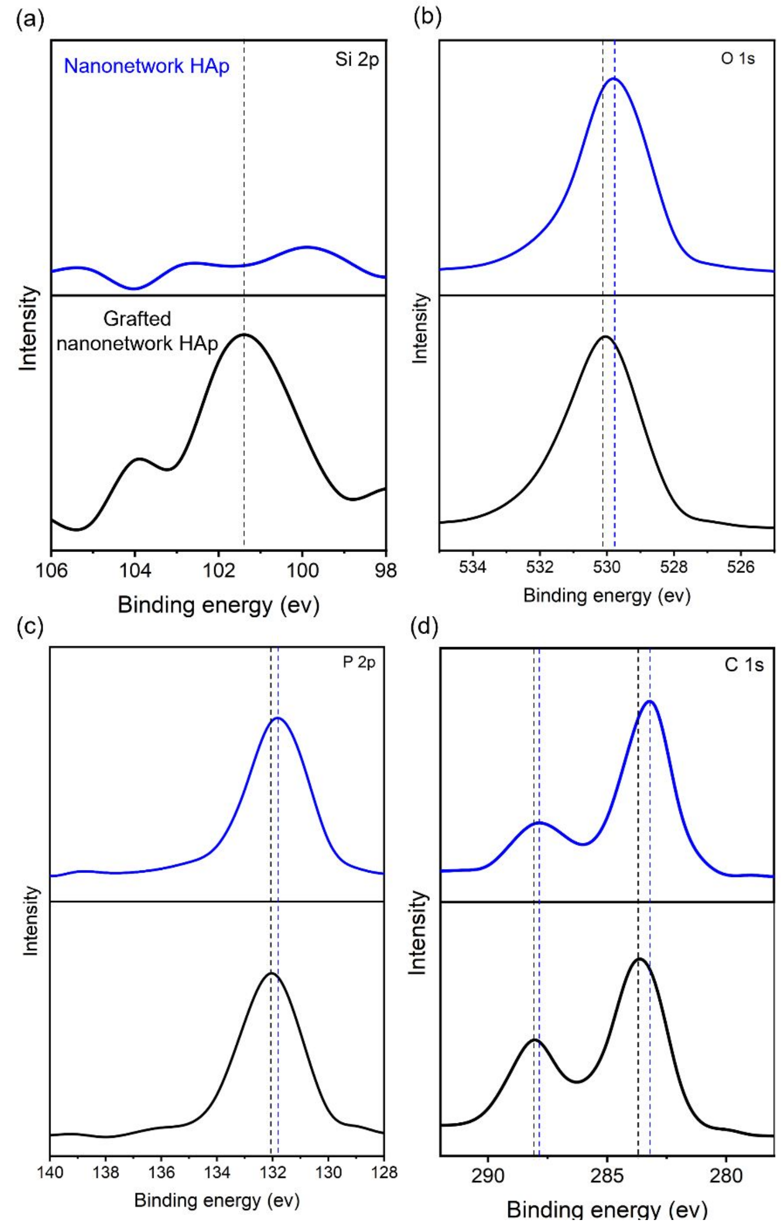


**Figure S3. Characterization of grafted diamond-structured HAp.** XPS spectra of non-grafted and grafted nanonetwork HAp of (a) Si 2p, (b) O 1s, (c) P 2p, and (d) C 1s. For non-grafted HAp, the characteristic peaks of C, P, and O elements are similar to previous studies.[5,6] The characteristic peaks of Si 2p can be clearly observed on the HAp surface, which indicates the presence of γ-MPS. Additionally, the binding energies of the C 1s, P 2p, and O1s shift to a higher position due to the chemical interaction between HAp and *γ*-MPS. Those shifts evidence the successful grafting of *γ*-MPS onto HAp.

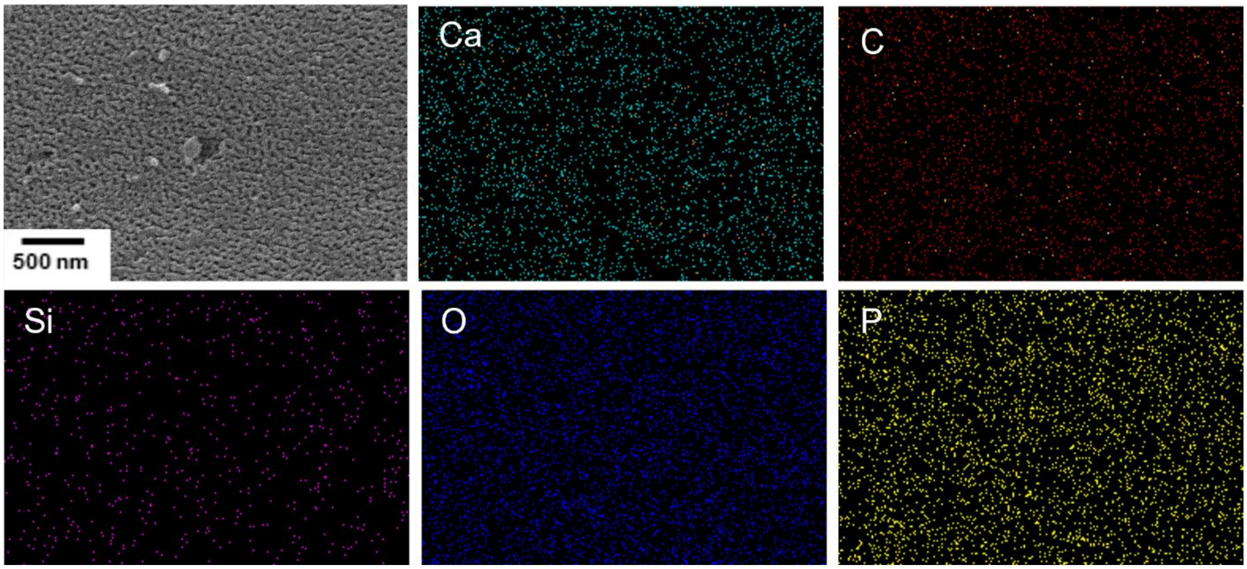


**Figure S4. EDS Mapping of PMMA/HAp nanohybrids**. FESEM micrograph of bicontinuous PMMA/HAp and corresponding EDS mapping analysis of the surface, showing the distribution of calcium, carbon, phosphorus, and oxygen elements from PMMA/HAp nanohybrids and silicon elements from *γ*-MPS, indicating the successful fabrication of the aimed PMMA/HAp nanohybrids

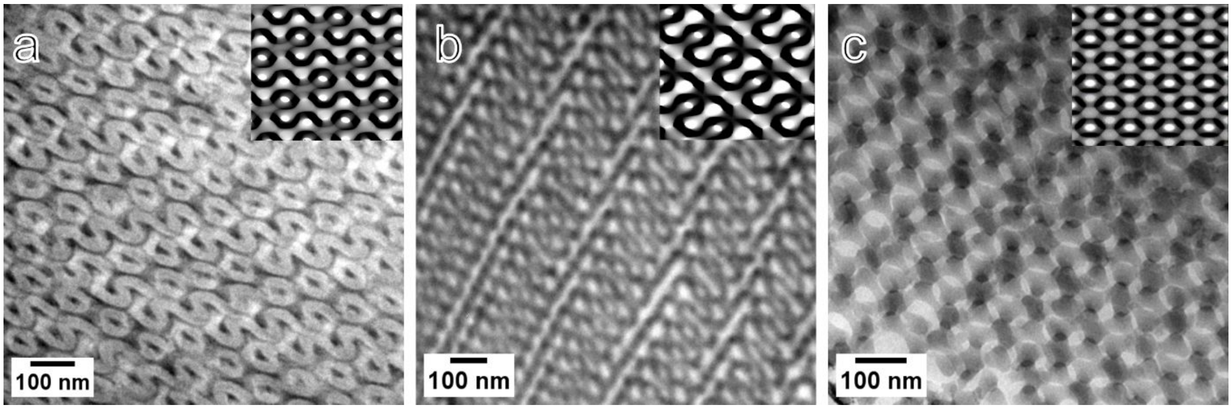


**Figure S5. Self-assembled PS-*b*-PDMS**. (a) TEM micrograph of diamond-structured PS-*b*-PDMS along [321] projection. (b) TEM micrograph of PS/HAp along [311] projection. (c) TEM micrograph of free-standing HAp nanonetwork after removal of PS template by calcination along [111] projection. Insets show the corresponding simulated TEM images.

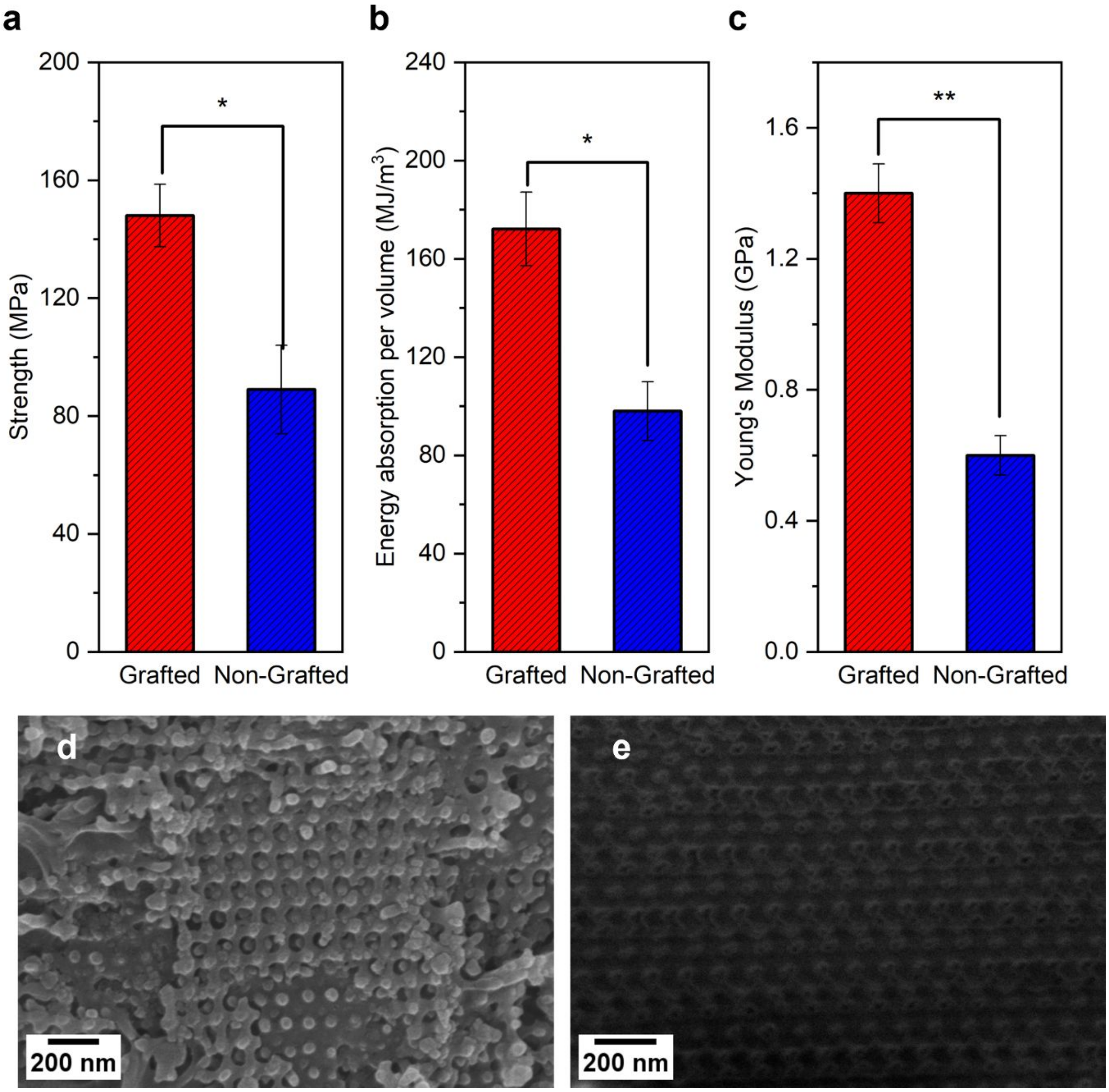


**Figure S6. Influence of interfacial bonding on mechanical performance and pore-filling efficiency.** Comparison of (a) strength, (b) energy absorption per volume, and (c) Young's modulus between grafted PMMA/HAp and non-grafted PMMA/HAp nanohybrids. Asterisks imply statistically significant differences. (d) FESEM of the fracture surface of non-grafted PMMA/HAp which leads to weaker boundaries and poor infiltration. (e) FESEM of grafted PMMA/HAp in which the chemical grafting gives rise to stronger boundaries, giving a smooth fracture surface.

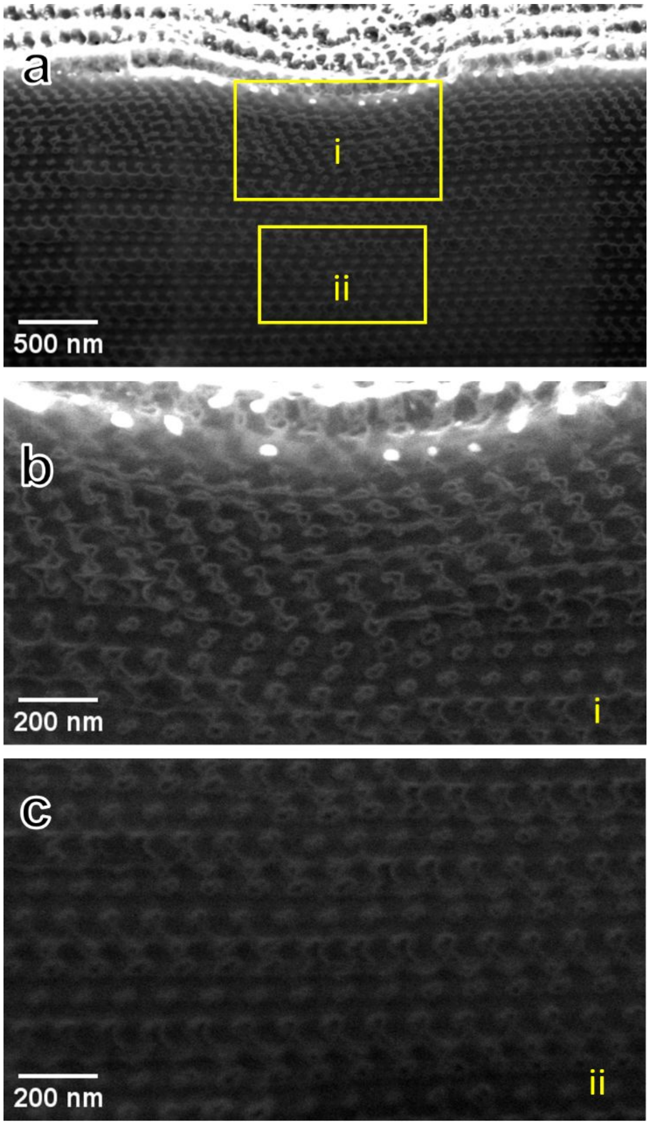


**Figure S7. Deformation of PMMA/HAp nanohybrids.** (a) Post-indentation FESEM micrograph of the underlying surfaces exposes the contact deformation of grafted PMMA/HAp nanohybrids. (b) High magnification FESEM micrograph of area located directly beneath the indenter showing high densification of HAp nanonetwork (white domain) embedded in the PMMA matrix (dark domain) that provides an additional toughening mechanism. (c) High magnification FESEM micrograph of the undeformed area that represents the surrounding region (yellow box (ii) in **a**).

## References


(1) Lo, T. Y.; Chao, C. C.; Ho, R. M.; Georgopanos, P.; Avgeropoulos, A.; Thomas, E. L. Phase Transitions of Polystyrene-b-Poly(Dimethylsiloxane) in Solvents of Varying Selectivity. *Macromolecules* **2013**, *46*, 7513–7524.

(2) Georgopanos, P.; Lo, T. Y.; Ho, R. M.; Avgeropoulos, A. Synthesis, Molecular Characterization and Self-Assembly of (PS-b-PDMS)n Type Linear (n = 1, 2) and Star (n = 3, 4) Block Copolymers. *Polym. Chem.* **2017**, *8*, 843–850.

(3) Lee, T.-L.; Lin, J.-W.; Ho, R.-M. Controlled Self-Assembly of Polystyrene- Block - Polydimethylsiloxane for Fabrication of Nanonetwork Silica Monoliths. *ACS Appl. Mater. Interfaces* **2022**, *14*, 54194–54202.

(4) Sadek, H.; K. Siddique, S.; Wang, C. W.; Lee, C. C.; Chang, S. Y.; Ho, R. M. Bioinspired Nanonetwork Hydroxyapatite from Block Copolymer Templated Synthesis for Mechanical Metamaterials. *ACS Nano* **2022**, *16* (11), 18298–18306.

(5) Yang, Z.; Huang, Y.; Chen, S. T.; Zhao, Y. Q.; Li, H. L.; Hu, Z. A. Template Synthesis of Highly Ordered Hydroxyapatite Nanowire Arrays. *J. Mater. Sci.* **2005**, *40*, 1121–1125.

(6) Zhao, H.; Liu, S.; Wei, Y.; Yue, Y.; Gao, M.; Li, Y.; Zeng, X.; Deng, X.; Kotov, N. A.; Guo, L.; Jiang, L. Multiscale Engineered Artificial Tooth Enamel. *Science* **2022**, *375*, 551–556.
